# Can the treatment effects of human-animal interaction be maintained? A randomized controlled trial including follow-up in people with severe mental illness

**DOI:** 10.3389/fpsyt.2026.1765531

**Published:** 2026-05-14

**Authors:** Petra Schmid, Stefan Tschöke, Carmen Nauss, Petra Prinz, Claudia Jauch-Ederer, Anna Lena Kordeuter, Carmen Uhlmann

**Affiliations:** 1 Klinik für Psychiatrie und Psychotherapie I Universität Ulm, Weissenau, Ravensburg-Weissenau, Germany; 2 ZfP Südwürttemberg, Versorgungsforschung, Ravensburg, Germany; 3 Prinzenhof, Leutkirch, Germany

**Keywords:** animal welfare, animal-assisted intervention, animal-assisted services, borderline personality disorder, emotion regulation, psychiatry, sheep, substance use disorder

## Abstract

**Introduction:**

There are persistent demands for well-designed randomized controlled trials (RCTs), including follow-up measurements, in studies on animal-assisted treatment (AAT). In addition, a possible dose-response relationship is under discussion. The aim of the present study was to investigate the efficacy of a single-session AAT with sheep, including a booster exercise, over a follow-up period of four weeks.

**Methods:**

In an RCT, a single-session AAT with sheep in a group setting, including an imaginative booster exercise conducted in the week following the AAT session, was compared to treatment as usual (TAU). Sixty psychiatric inpatients with severe mental illness were assessed for positive and negative emotions, mindfulness, and self-efficacy expectancy at baseline (PRE), immediately after the intervention (POST), and at one-week and four-week follow-ups.

**Results:**

The results indicate significant differences between the two groups at POST and still in the one-week follow-up (FU1) in three of four outcomes. Within the intervention group, within-group analyses demonstrated significant improvements from PRE to POST and from PRE to FU1 across all outcomes, with large effect sizes. At the four-week follow-up, all significant effects had diminished.

**Conclusions:**

An imaginative booster exercise conducted within one week after an AAT session was effective in maintaining large effect sizes for up to one week. However, the results did not persist at the four-week follow-up. Longer follow-up periods, variations in the number of sessions, and the inclusion of active control groups are therefore necessary for further AAT studies.

**Trial registration:**

https://drks.de/search/de/trial/DRKS00031347, identifier DRKS 00031347

## Introduction

The inclusion of animals in therapeutic interventions has a long tradition (1). Professionalization has increased over the years, especially in ethical terms (2, 3). However, methodologically, there are persistent demands for well-designed RCTs with sample size calculations, well-chosen outcome measures, effect size estimates, and follow-up measurements (e.g. 4, 5). Part of this development is also the initiative of Binder and colleagues (6), which resulted in a consensus-based revised definition of animal-assisted interventions. The term animal-assisted services (AAS) covers the entire spectrum of practices in which animals are included in various roles for the benefit of humans. Depending on the focus, this main category is further subdivided into animal-assisted support programs (AASP), animal-assisted education (AAE), and animal-assisted treatment (AATx). AATx refers to a range of treatment methods for people with mental or physical disorders that include animals. A key feature of AATx is that it is performed by licensed professionals with additional training in AATx and serves as an adjunct to another treatment approach (6).

The efficacy of methodologically high-quality AAS studies has been examined in several reviews. These reviews demonstrate positive effects of AAS with various animals across a wide variety of samples, age groups and mental disorders (depression, schizophrenia, autism, PTSD) using different outcome measures (e.g. 5, 7–13). Therefore, the efficacy of AAS can be assumed. An early large meta-analysis of AAT, which included 49 studies, concluded that AAS is effective, but that different conditions do not significantly influence the outcome (14). These conditions include the type of animal species, type of participants, number of sessions, type of intervention, type of outcomes, and study design. Some important findings were that younger participants benefited more, and non-disabled individuals displayed stronger and more reliable benefits in terms of changes in emotional well-being and behavior. Individual AAT was more beneficial than group AAS.

Another question concerns the underlying mechanism of AAT’s efficacy. This has been investigated in four studies. Various theories and hypotheses are currently being discussed, such as providing experiential learning including co-regulation of emotional states; supporting activation; developing alternative relationships or mediating social interactions; offering novel ways of processing thoughts and emotions; collaborating in therapeutic processes; enhancing self-efficacy; or improving well-being (15–18). In accordance to this, Wagner and colleagues (19) identified human-animal interaction, relationships, and social or emotional support in their systematic review of the mechanisms of action of AAT. To date, no clear conclusions can be drawn regarding the differential inclusion of animal species in terms of efficacy or mechanisms of action. Whereas dogs were primarily involved in research in the past (14), sheep also appear to be suitable in AAT. Sheep are well suited for the inclusion in AAT due to their nature as flight animals, as well as f their social, emotional, and communicative characteristics (20, 21). For successful AAT, it seems important to pay attention to the needs of sheep and to acknowledge them respectfully, which makes sheep particularly suitable for mindfulness-based interventions (22).

Although the efficacy of AAT appears to be evident, it remains unclear whether the effects persist over a longer period of time. Only a few of the studies published to date include follow-up measurements. For example, in a systematic review of AAS in mental health treatment for adolescents, none of the seven included studies conducted follow-up examinations (10). Similar results were found in a systematic review of AAS in psychiatric inpatients (12) and in people with intellectual disabilities (11). Likewise, in a review of the efficacy of AAS in children and adults with symptoms of post-traumatic stress disorder, 41 studies were included, but only five studies contained follow-up data (9). A similar picture emerges in the systematic review of RCTs of AAT in psychiatric patients (5). Of the total of seven included RCTs, only two studies collected follow-up data, both on AAT with farm animals. In the first study, the 29 depressed participants achieved a significant reduction in depression and improvement in self-efficacy over the course of the 12-week intervention and were able to maintain this effects in the three-month follow-up, in contrast to the control group (23). In the second study, involving 90 participants with psychiatric disorders, improvements in the self-efficacy and coping abilities in AAT group were not evident immediately after the treatment but emerged in the six-months follow-up. On the other hand, depression scores displayed improvement after six months in the AAT group, but also in the control group (24, 25). In sum, there is evidence of the efficacy of AAS, but there is still uncertainty about the persistence of these effects. Accordingly, the authors concluded that it is crucial to conduct follow-up studies, as it is important to determine how long the effects of AAT last (5).

Our research focuses on mindfulness-based AAT with sheep in psychiatric patients with emotion regulation disorders. In an initial study, the effects of a single AAT session with sheep were examined using a controlled design with a follow-up period of up to one week in individuals with severe mental illness (SMI) (26). A total of 36 participants diagnosed with a substance use disorder (SUD) and comorbid diagnoses were included. Compared to the control group receiving treatment as usual, significant improvements in positive and negative emotions, mindfulness, and self-efficacy expectancy were observed in the AAT group. However, at the one-week follow-up, these effects were no longer detectable, and no differences between the two groups remained. The qualitative analysis demonstrated that mindfulness and positive emotional valence were crucial factors in the efficacy of AAT (26). In a replication study, 29 psychiatric inpatients with SUD and another group of 31 patients with borderline personality disorder (BPD) were examined in an RCT (27). The promising results of the initial study were replicated, with improvements in the aforementioned outcomes directly after the intervention and in contrast to the control group. It was also demonstrated that the effects were similar in both disorders (SUD and BPD). This time, in addition to the single session AAT with sheep, an imaginative booster exercise to evoke mindfulness and positive emotional valence was conducted in the week following the AAT session. This was implemented to reactivate and consolidate the emotional experience of the AAT session retrospectively. Pile and colleagues (28) defined emotional mental imagery as the ability to simulate and manipulate experiences with multiple sensory input. Imagery is assumed to change behavior by influencing internal states, improving self-efficacy, and reducing anxiety (29).

The aim of the present study was to investigate the efficacy of a single AAT session with sheep, including an imaginative booster exercise, over a longer period of time. For this purpose, participants receiving treatment as usual (TAU) were compared with participants receiving additional AAT (TAU+AAT) in a repeated-measures design with a follow-up period of four weeks. The main hypothesis was therefore that participants in the TAU+AAT group would show significantly greater improvements in the primary outcomes (positive and negative emotions sum score) over a follow-up period of four weeks, with four measurement points, compared to participants in the TAU group (group differences over time). Furthermore, participants in the TAU+AAT group were expected to show significantly greater improvements in the secondary outcomes (mindfulness and self-efficacy expectancy) over the same follow-up period compared to participants in the TAU group (group differences over time).

## Materials and methods

### Study design

A randomized controlled, repeated-measure trial was conducted comparing treatment as usual (TAU) with a protocol-driven AAT in addition to TAU (TAU+AAT). A total of sixty psychiatric inpatients were examined before (PRE), after (POST), one week after (FU1), and one month after (FU2) the intervention, with the same measuring time points applied to the TAU group. Results comparing the two groups (TAU vs TAU+AAT) at PRE and POST have been published elsewhere (27).

Ethical approval was obtained from the ethics committee of the University of Ulm (45/23). In accordance with the Declaration of Helsinki, participants were informed and provided written informed consent. The study was registered in the German Registry for Clinical Studies (DRKS00031347, date of first registration April 20, 2023).

### Participants

**Figure 1:**
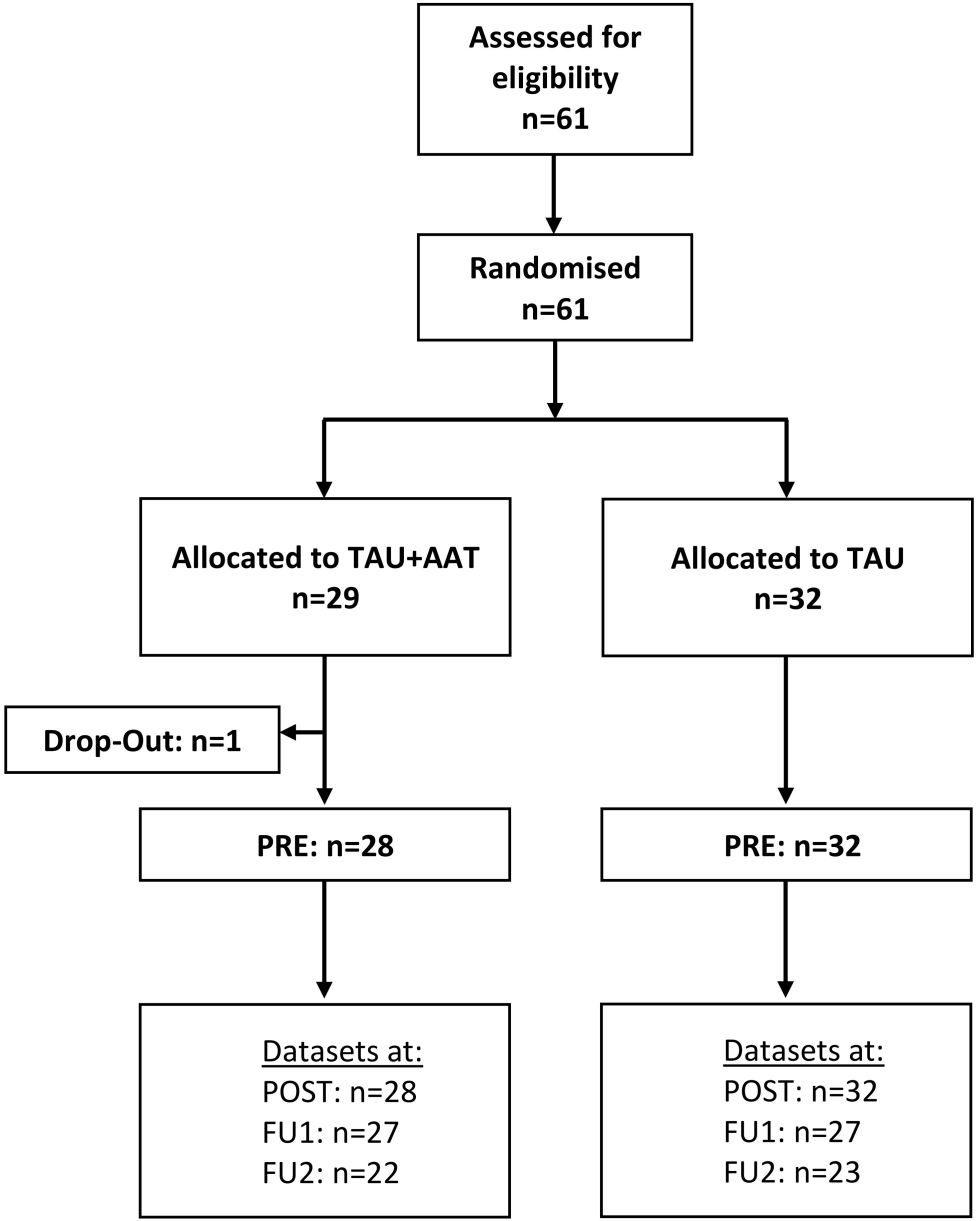
CONSORT for the recruitment of the treatment as usual (TAU) group and the group with additional animal-assisted treatment (TAU+AAT) and the number of datasets at PRE, POST, Follow-Up 1 (FU1), and Follow-Up 2 (FU2).

Psychiatric inpatients at a university hospital for psychiatry and psychotherapy in Germany with a severe mental illness (SMI), specifically a primary diagnosis of substance use disorder (SUD) or borderline personality disorder (BPD), were examined. Inclusion criteria were age between 18 and 65 years, an SMI requiring inpatient treatment lasting at least seven days, the ability to provide informed consent, and physical ability to stand and walk safely. Exclusion criteria were animal phobia, allergies, or aversion to animals (30) and acute psychosis. After providing informed consent, eligible participants were randomized to either the TAU+AAT group or the TAU group. The CONSORT flow diagram, including available datasets is shown in **Figure 1**.

### Intervention: AAT session, animal welfare, and imaginative booster exercise

The AAT session took place at a farm specialized in AAT in southern Germany (“Prinzenhof”) and was conducted by three certified specialists: PP (responsible for the animal welfare and supervision of the sheep), CN (responsible for conducting the AAT procedure), and another additional licensed therapist. This therapist ensured participants’ well-being and facilitated the transfer of therapeutic experiences from the AAT session into the ongoing psychotherapeutic inpatient treatment (see 26, 27 for details). The protocol-driven AAT consisted of a single group session with sheep and lasted approximately 5 hours, including travel time. The procedure included seven sections: 1. Observation of PP´s interaction with the sheep, 2. Introduction of the sheep, 3. Approach via feeding, 4. Approach via presence, 5. Experience of competence and attachment, 6. Free walk in mindful interaction, and 7. Farewell. Each group consisted of four participants and four sheep. For a detailed description of the AAT procedure and the included sheep, see Schmid and colleagues (27). Regarding animal welfare, the intervention was based on the One Health framework, which considers human, animal, and environmental health (3). The study followed established quality standards for animal-assisted services (31–35) and an adapted risk assessment tool for animal-human interaction (36).

In addition, an imaginative booster exercise was conducted within one week after the AAT session. This guided imagery exercise (28), lasting 30 minutes and conducted individually, aimed to consolidate the emotional and mindful experiences from the AAT session. Participants were instructed to reactivate these experiences in future situations as needed.

### Materials

The primary outcome of positive and negative emotions was measured using the State-Trait Anxiety Inventory - State version (STAI-S; 37, 38) at PRE, POST, FU1, and FU2. The STAI-S includes 20 items rated on a four-point Likert scale (1 = not at all to 4 = very much so). Internal consistency is high (Cronbach’s alpha = .92-.94). Two indices (positive and negative emotions sum score) were calculated, with higher values indicating higher levels of the respective construct.

Secondary outcomes included mindfulness and self-efficacy expectancy. Mindfulness was assessed using Freiburg Mindfulness Inventory (FMI, short form; 39, 40). The 14-item scale uses a four-point Likert scale format (1 = rarely to 4 = almost always) and yields a sum score. With a McDonald’s omega of .87, the scale demonstrates good internal consistency (40). Perceived self-efficacy was measured using the General Self-Efficacy Short Scale (ASKU; 41), consisting of three items rated on a five-point Likert scale (1 = doesn’t apply at all to 5 = applies completely). The mean score used for analysis. Good internal consistency is also reported for this scale, with a McDonald’s omega of .81 and .86 (41). All secondary outcomes were assessed at PRE, POST, FU1, and FU2. In both questionnaires, higher values indicate a higher level of mindfulness or self-efficacy expectancy.

Social integration was measured using the Objective Social Outcome Index (SIX; 42), covering employment, accommodation, partnership/family, and friendships. Scores range from 0 to 6, with higher scores indicating better integration. In addition, sociodemographic (age, gender) and clinical meter data (diagnoses, duration of inpatient treatment) were extracted retrospectively from medical records.

### Power calculation

The previous study (26) showed an average effect sizes of r = 0.55 for the STAI-S sum score between the two groups. At FU1, no between group effect remained. Using G*Power 3.1 for a two-group (TAU+AAT vs TAU), four-time-point (PRE, POST, FU1, FU2) repeated-measure ANOVA with an average effect size of r = 0.55/2 resulting in r = 0.27, 1-ß = .95, alpha = .05, a required sample size of n = 56 complete datasets was calculated.

### Statistical analyses

Analyses were conducted using IBM SPSS 27^®^. If fewer than 10% of values per questionnaire were missing (which was the case for n = 9 participants), imputation was applied using the mean value. Chi-square tests were performed to examine group differences in dichotomous variables. Normal distribution of the data was checked using the Kolmogorov-Smirnov test. For non-normally distributed data, group differences were analyzed using the Mann-Whitney U test.

Repeated-measures ANOVAs were calculated for all primary and secondary outcomes. Despite minor violations of normality, ANOVA was retained due to robustness (43, violations in STAI-S positive emotions sum score at PRE and FU2, in FFA sum score at PRE and POST, in ASKU mean value at POST). Sphericity (Mauchly´s test) was not violated. Cohen’s d was calculated for effect size with d<.5 representing a small effect size, .5<d<.8 representing a moderate effect size and d>.8 representing a large effect (44). For a detailed examination of time effects, *post hoc* Wilcoxon tests were calculated for participants in the AAT+TAU group. Mann-Whitney U tests were calculated to examine *post hoc* group differences at PRE, POST, FU1, and FU2. *post hoc* analyze No Bonferroni correction was applied due to the exploratory nature of the *post-hoc* analyses. In both post-hoc analyses, the correlation coefficient r is calculated for effect sizes, whereby according to Cohen (44) r< .3 is regarded as a small effect, .3<r<.5 as a medium effect and r>.5 as a large effect.

## Results

### Sociodemographic and clinical description of the sample

The n=60 inpatient psychiatric participants with a primary diagnosis of substance use disorder (SUD) or borderline personality disorder (BPD), were, on average, 34.90 years old (SD = 12.54). Of the participants, 71.7% were female and 28.3% male; there were no non-binary participants. Participants received inpatient treatment for an average of 50.20 days (SD = 61.41). The social integration index (SIX) averaged 3.40 (SD = 1.55) and the mean number of additional diagnoses was n=4.03 (SD = 3.54).

The comparability of the TAU+AAT and TAU groups was examined at PRE. No statistically significant differences were found in any of the demographic or clinical variables, indicating that the two groups can be considered comparable (see **Table 1**).

**Table 1:** Group comparison of participants receiving additional animal-assisted treatment (TAU+AAT) versus participants receiving treatment as usual (TAU).

Participants			TAU+AAT n=28		TAU n=32		P
age		M (SD)	35.82	(13.01)	34.09	(12.27)	n.sign.^1^
sex	female	n (%)	18	(64.3%)	25	(78.1%)	n.sign.^2^
	male	n (%)	10	(35.7%)	7	(21.9%)	
number of additional diagnoses		M (SD)	4.71	(4.05)	3.44	(2.96)	n.sign.^1^
duration of stay (in days)		M (SD)	59.54	(85.09)	42.03	(26.90)	n.sign.^1^
social integration (SIX)		M (SD)	3.46	(1.37)	3.34	(1.72)	n.sign.^1^

SIX, Objective Social Outcome Index; ^1^Mann-Whitney-U-test; ^2^Chi²-test.

### Question 1: between-and within-group effects up to one-week follow-up

For the primary outcomes (STAI-S negative emotion sum score and STAI-S positive emotion sum score) a significant time x group interaction emerged, with large effect sizes (STAI-S negative emotions sum score: F(2,51)=9.975; p<.001; Cohen’s d=1.04; STAI-S positive emotions sum score: F(2,51)=37.083; p<.001; Cohen’s d=2.41). Significant time x group interactions with large effect sizes were also observed in the two secondary outcomes mindfulness and self-efficacy expectancy (FMI sum score (F(2,52)=17.908; p<.001; Cohen’s d=1.66; ASKU mean value F(2,52)=11.938; p<.001; Cohen’s d=1.36).

**Figure 2:**
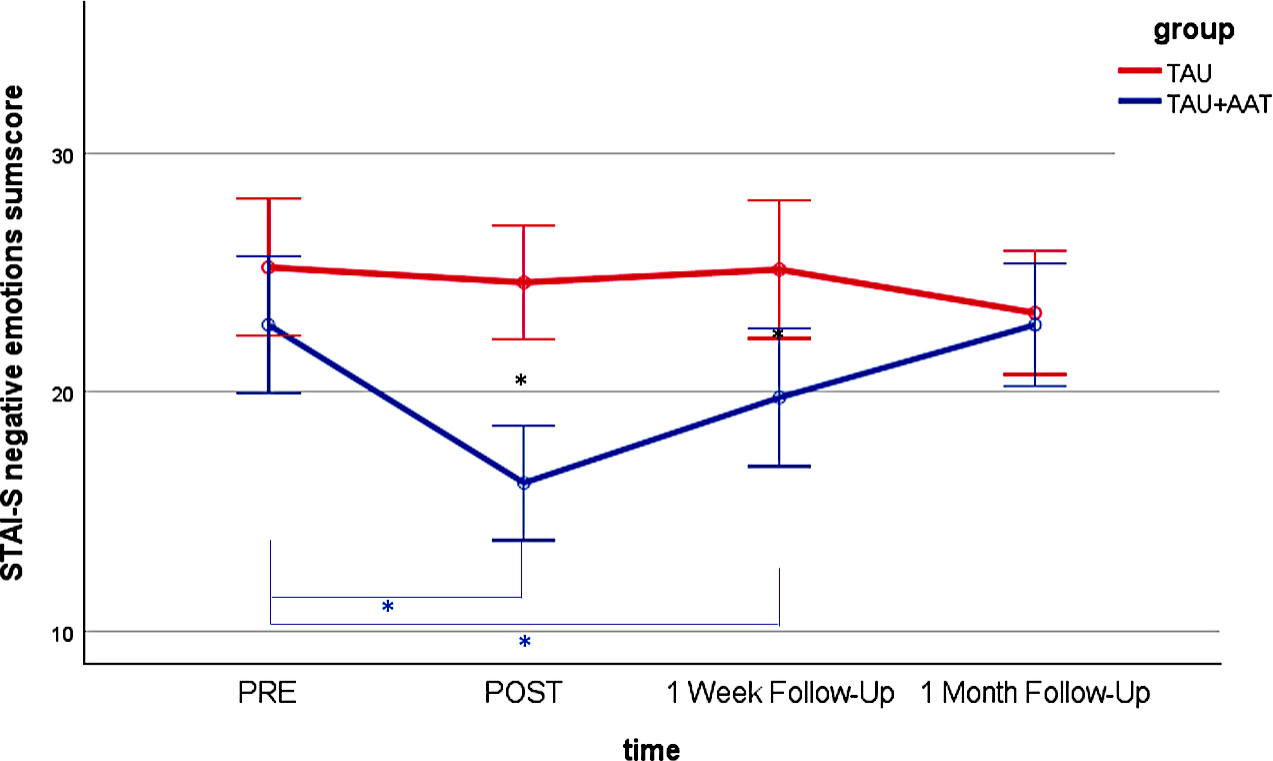
STAI-S negative emotions sum score over time (PRE, POST, 1 Week Follow-Up, 1 Month Follow-Up) for both groups (TAU+AAT vs TAU) including significant time effects in TAU+AAT group (blue asterisk), and significant group effects (black asterisk). The error bars represent the 95% confidence intervals.

**Figure 3:**
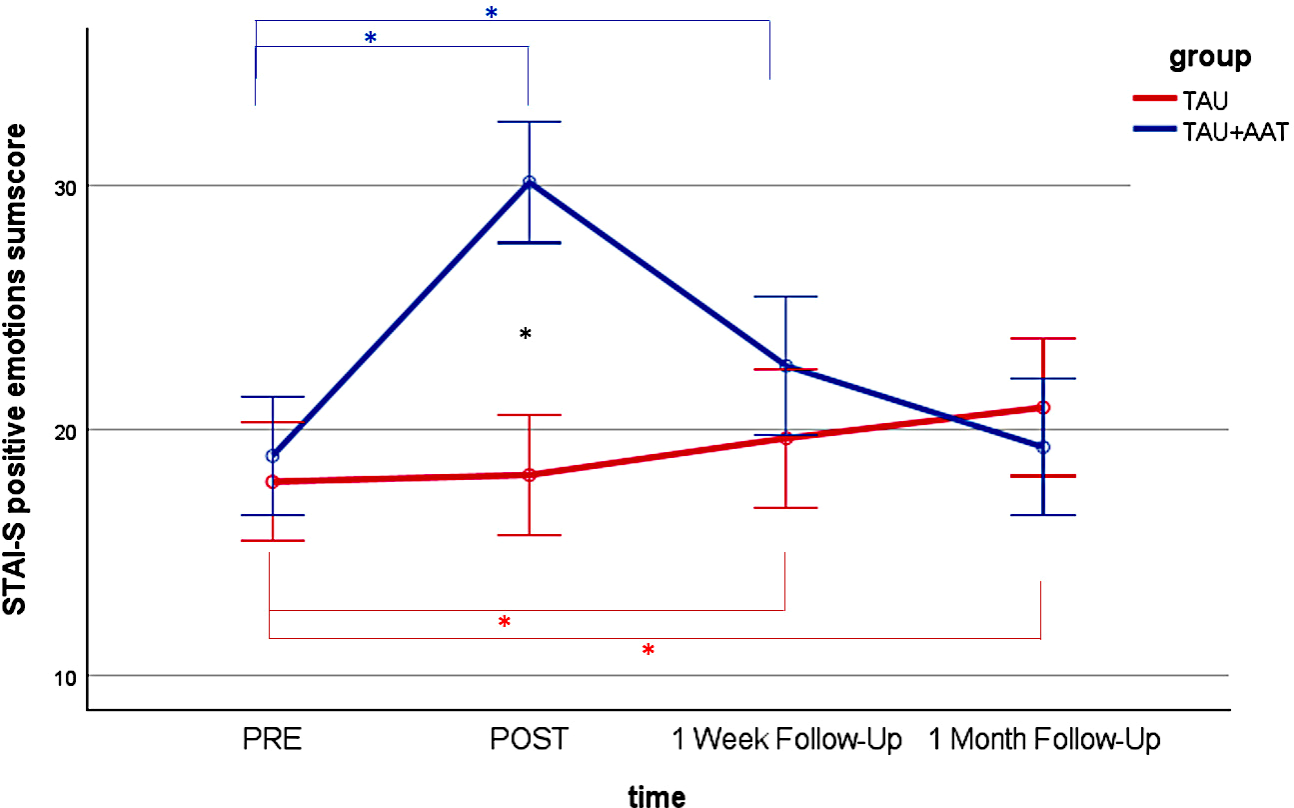
STAI-S positive emotions sum score over time (PRE, POST, 1 Week Follow-Up, 1 Month Follow-Up) for both groups (TAU+AAT vs TAU) including significant effects in TAU+AAT group (blue asterisk), significant time effects in TAU group (red asterisk), and significant group effects (black asterisk). The error bars represent the 95% confidence intervals.

**Figure 4:**
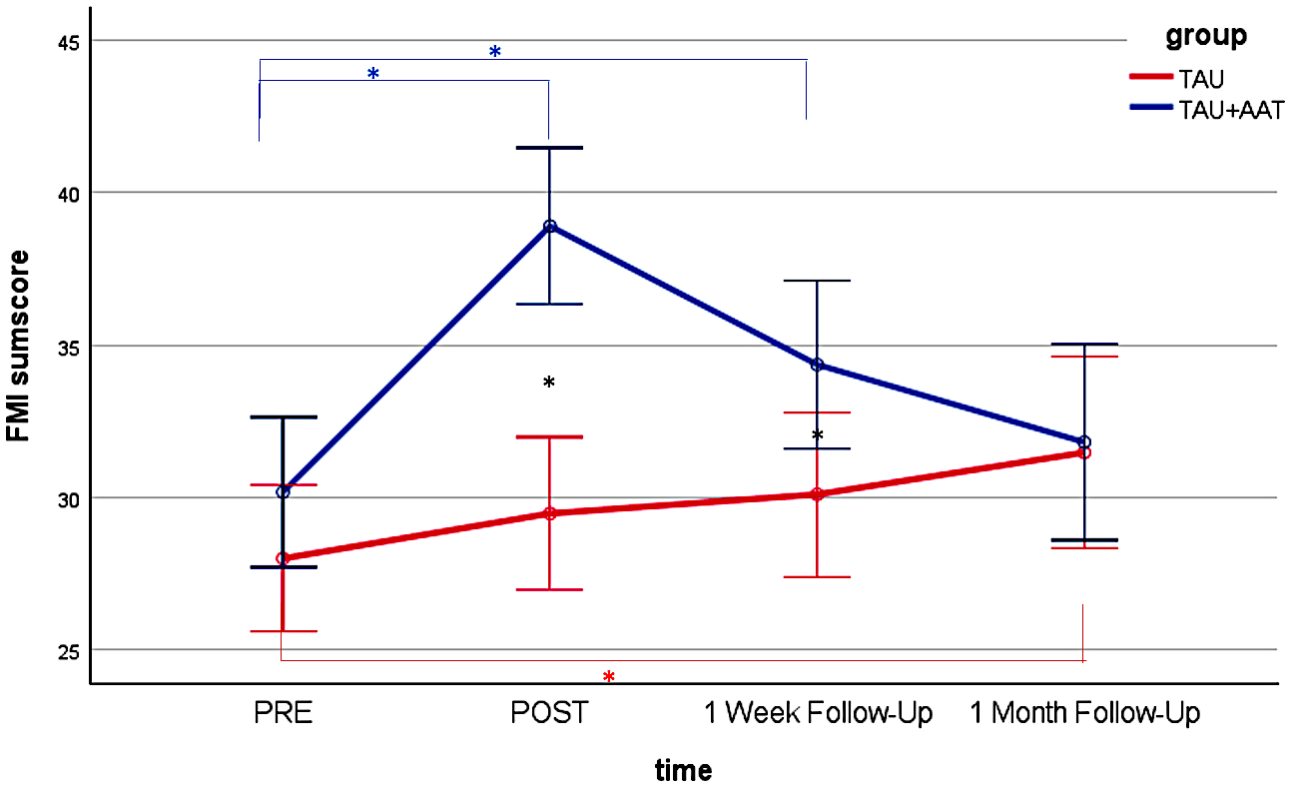
FMI sum score over time (PRE, POST, 1 Week Follow-Up, 1 Month Follow-Up) for both groups (TAU+AAT vs TAU) including significant time effects in TAU+AAT group (blue asterisk), significant time effects in TAU group (red asterisk), and significant group effects (black asterisk). The error bars represent the 95% confidence intervals.

**Figure 5:**
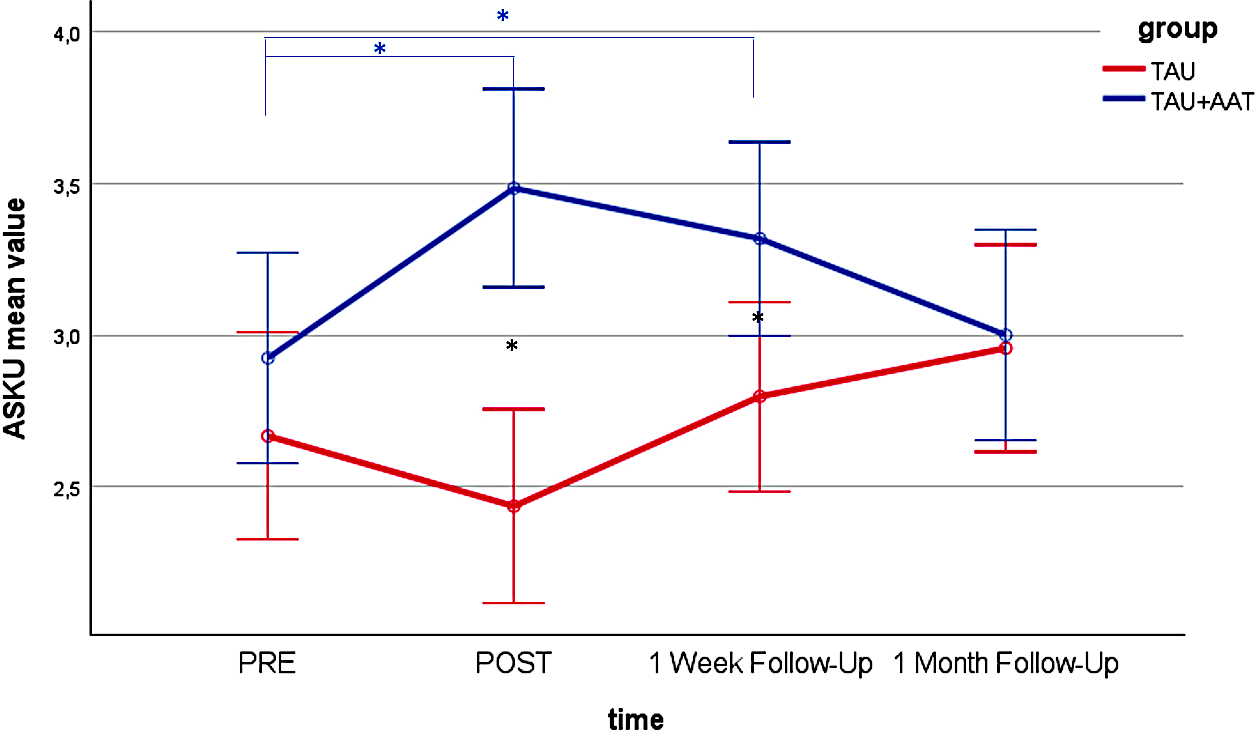
ASKU mean value over time (PRE, POST, 1 Week Follow-Up, 1 Month Follow-Up) for both groups (TAU+AAT vs TAU) including significant time effects in TAU+AAT group (blue asterisk), and significant group effects (black asterisk). The error bars represent the 95% confidence intervals.

*Post hoc* analyses of the between-group comparisons showed no differences between the two groups at PRE in all four outcomes. Significant differences with large effect sizes were observed at POST (STAI-S negative emotions sum score: p<.001; Cohen’s r=.631; STAI-S positive emotions sum score: p<.001; Cohen’s r=.738; FMI sum score: p<.001, r=.647; ASKU mean value: p<.001; Cohen’s r=.570) and at FU1 (STAI-S negative emotions sum score: p<.01; Cohen’s r=.369; FMI sum score: p<.05; Cohen’s r=.299; ASKU mean value: p<.05; Cohen’s r=.311) with medium effect sizes, each favoring the TAU+AAT group. **Figures 2**–**5** illustrate these effects.

In the within-group comparisons, participants in the TAU+AAT group improved significantly between PRE and POST in all four outcomes, each with large effect sizes (STAI-S negative emotions sum score: p<.001; Cohen’s r=.853; STAI-S positive emotions sum score: p<.001; Cohen’s r=.859; FMI sum score: p<.001; Cohen’s r=.850; ASKU mean value: p<.001; Cohen’s r=.680). Between PRE and FU1, significant effects were evident in all four outcomes, again with large effect sizes (STAI-S negative emotions sum score: p<.01; Cohen’s r=.555; STAI-S positive emotions sum score: p<.01; Cohen’s r=.540; FMI sum score: p<.01; Cohen’s r=. 508; ASKU mean value: p<.01; Cohen’s r=.594). Within the TAU group, comparisons between PRE and POST and between PRE and FU1 were not significant, except for STAI-S positive sum score between PRE and FU1 (p<.05; Cohen’s r=.443). **Figures 2**–**5**, **Table 2** illustrate these effects.

**Table 2:** Means and standard deviations of the four outcomes for participants receiving additional animal-assisted treatment (TAU+AAT) and participants receiving treatment as usual (TAU) at PRE, POST, 1 Week Follow-Up, and 1 Month Follow-Up.

Outcome	Group	PRE	POST	1 Week Follow-Up	1 Month Follow-Up
		M (SD)	M (SD)	M (SD)	M (SD)
STAI-S negative emotions sum score	TAU+AAT	22.82 (7.18)	16.19 (5.18)	19.77 (6.75)	22.82 (6.88)
	TAU	25.23 (6.10)	24.60 (5.96)	25.14 (6.63)	23.32 (4.97)
STAI-S positive emotions sum score	TAU+AAT	18.96 (6.52)	30.10 (6.51)	22.62 (7.59)	19.32 (7.00)
	TAU	17.91 (4.52)	18.18 (4.82)	19.68 (5.31)	20.93 (5.92)
FMI sum score	TAU+AAT	30.18 (6.48)	38.91 (7.05)	34.36 (7.81)	31.82 (8.75)
	TAU	28.00 (4.88)	29.48 (4.77)	30.10 (4.69)	31.48 (5.99)
ASKU mean value	TAU+AAT	2.92 (0.79)	3.48 (0.64)	3.32 (0.79)	3.00 (0.88)
	TAU	2.67 (0.83)	2.43 (0.86)	2.80 (0.69)	2.96 (0.74)

### Question 2: between-and within-group effects up to one-month follow-up

Taking all four time points into account, significant time x group interactions were observed for all four outcomes, with large effect sizes (STAI-S negative emotions sum score: F(3,40)=8.637; p<.001; Cohen’s d= 1.61; STAI-S positive emotions sum score: F(3,40)=21.664; p<.001; Cohen’s d=2.55; FMI sum score: F(3,41)=11.261; p<.001; Cohen’s d=1.82; ASKU mean value: F(3,41)=9.217; p<.001; Cohen’s d=1.64).

In *post hoc* analyses of the between-group comparisons, the two groups no longer differed significantly at FU2 in any outcome. In within-group analyses, there were no significant differences in the TAU+AAT group between PRE and FU2 across all four outcomes. However, in the TAU group, significant differences between PRE and FU2 were observed in two outcomes (STAI-S positive emotions sum score: p<.05; Cohen’s r=.451; FMI sum score: p<.05; Cohen’s r=.417). **Figures 2**–**5**, **Table 2** illustrate the effects described.

## Discussion

The aim of the present study was to investigate the efficacy of a single-session AAT with sheep as add-on to psychiatric inpatient treatment (TAU+AAT). This was examined in a repeated-measures RCT design compared to TAU over a follow-up period of four weeks. To maintain the therapeutic impact of the AAT session, the intervention was enhanced with an imaginative booster exercise conducted within one week after the AAT session.

The results indicate significant differences between the intervention and the control group directly after the AAT session (POST) in all four outcomes, with large effect sizes, and still in the one-week follow-up in negative emotions, mindfulness, and self-efficacy expectancy, with medium effect sizes. Within the intervention group, within-group analyses demonstrated significant improvements in all outcomes from PRE to POST and in three outcomes from PRE to one-week follow-up. At the one-month Follow-up (FU2), all significant effects had diminished, both between the groups and within the intervention group.

The significant improvements observed in the present study were maintained for up to one week, which contrasts with our initial study (26). This effect is attributed to the imaginative booster exercise. The newly implemented imaginative booster exercise within one week after the AAT session aimed to consolidate positive experiences by mindfully recalling interactions with the sheep, particularly with regard to positive emotions and self-efficacy. A similar approach to consolidating AAT effects during follow-up has been described in the work of another research group. Three months after completing an eight-week AAT program with sheep in a group setting, an additional booster session with the sheep was conducted. At the one-year follow-up, sustained improvements were observed in the AAT group across various outcomes compared to TAU. However, the extent to which these sustained effects can be attributed to the booster session remains unclear (22).

Nevertheless, our findings suggest that a booster session can enhance short-term sustainability, as the initial study without a booster showed only minor effects after one week. In contrast, the present study demonstrated maintained effects up to one week (FU1), although not beyond this time point.

As noted above, no significant effects were observed in the one- month follow-up, neither between the groups nor within the intervention group (TAU+AAT). However, the control group (TAU) showed significant improvements in positive emotions and mindfulness, as has also been reported in other AAT studies (e.g., 24). It should be considered that the control group received standard psychiatric treatment and that participants in both groups may have continued inpatient treatment until the one-month follow-up. Another possible explanation is that, within the intervention group, the transfer of therapeutic experiences of the AAT into patients’ everyday lives - and thus the maintenance of the treatment effects - was not fully successful. This transfer may depend on the number of sessions. In their review, Maujean and colleagues (5) refer to a dose effect, suggesting that a longer duration of AAT may be associated with better results. Both Schramm and colleagues (22) and Pedersen and colleagues (23) conducted 8 and 12 sessions, respectively, with sheep. While Schramm and colleagues (22) reported sustained effects at the one-year follow-up, Pedersen and colleagues (23) found only partially maintenance at the three-month follow-up. However, the latter study has several methodological limitations (e.g., baseline group differences, small follow-up sample, waitinglist control condition, last observation carried forward in follow-up), which complicate interpretation. Other settings using different animal species have also demonstrated sustained effects, particularly when AAT is delivered continuously. For example, a study involving cats and dogs in a psychogeriatric ward with chronically schizophrenic patients showed that a weekly three-hour sessions significantly improved social functioning over one year when the intervention was maintained throughout that period (17). As outlined in the introduction, these findings provide substantial evidence for the efficacy of AAT. However, it remains unclear whether specific effects depend on participant characteristics, animal species, or other factors (14). Overall, there is currently insufficient evidence regarding both a potential dose effect of AAT and the long-term maintenance of its effects in follow-up assessments.

Our study has several limitations. One concerns limited generalizability as the intervention involved specific sheep, therapists, and participants. At the same time, efforts were made to enhance generalizability by using a manual-driven protocol with a detailed description of the intervention, while characteristics the sheep and therapists qualifications have been reported elsewhere (27). Additionally, some participants were lost to follow-up at FU2 (TAU: n = 9, TAU+AAT: n = 6). However, since attrition occurred in both groups within a randomized design, this limitation is unlikely to have systematically biased the results. Another limitation is the relatively short follow-up period of one month. Longer follow-up intervals (e.g., at least six months) as used in other studies (24, 25) would have been desirable.

## Conclusions

In conclusion, the limited generalizability and relatively small sample size should be considered when interpreting the findings. Nevertheless, the results contribute meaningfully to the literature. The imaginative booster exercise successfully maintained treatment effects for one week. The intervention also demonstrates high clinical feasibility and practicality, particularly because the booster session consists of a simple imaginative exercise. Given that the effects did not persist beyond four weeks, future research should examine whether increasing the number of sessions could enhance long-term outcomes and better account for potential dose effects. Accordingly, longer follow-up periods and the inclusion of active control groups are essential for advancing the methodology of further AAT studies.

## Data Availability

The raw data supporting the conclusions of this article will be made available by the authors, without undue reservation.

## References

[1] Seeger L Kübler A Hilger K . Drop-out rates in animal-assisted psychotherapy - results of a quantitative meta-analysis. Br J Clin Psychol. (2025) 64:166–87. doi: 10.1111/bjc.12492. PMID: 39101511 PMC12057310

[2] Chalmers D Dell CA . Applying one health to the study of animal-assisted interventions. Ecohealth. (2015) 12:560–2. doi: 10.1007/s10393-015-1042-3. PMID: 26063040 PMC4703413

[3] Hediger K Meisser A Zinsstag J . A one health research framework for animal-assisted interventions. Int J Environ Res Public Health. (2019) 16:640. doi: 10.3390/ijerph16040640. PMID: 30795602 PMC6406415

[4] Fornefeld D Zellin U Schmidt P Fricke O . The supporting role of dogs in the inpatient setting: a systematic review of the therapeutic effects of animal-assisted therapy with dogs for children and adolescents in an inpatient setting. Eur Child Adolesc Psychiatry. (2025) 34:3–17. doi: 10.1007/s00787-023-02326-1. PMID: 38147109 PMC11805780

[5] Maujean A Pepping CA Kendall E . A systematic review of randomized controlled trials of animal-assisted therapy on psychosocial outcomes. Anthrozoös. (2015) 28:23–36. doi: 10.2752/089279315X14129350721812

[6] Binder AJ Parish-Plass N Kirby M Winkle M Skwerer DP Ackerman L . Recommendations for uniform terminology in animal-assisted services (AAS). Human-Animal Interact. (2024) 12:1. doi: 10.1079/hai.2024.0003. PMID: 41771589

[7] Santaniello A Dicé F Carratú RC Amato A Fioretti A Menna LF . Methodological and terminological issues in animal-assisted interventions: an umbrella review of systematic reviews. Animals. (2020) 10:759. doi: 10.3390/ani10050759. PMID: 32349351 PMC7277107

[8] Villarreal-Zegarra D Yllescas-Panta T Malaquias-Obregon S Dámaso-Román A Mayo-Puchoc N . Effectiveness of animal-assisted therapy and pet-robot interventions in reducing depressive symptoms among older adults: a systematic review and meta-analysis. Complement Ther Med. (2024) 80:103023. doi: 10.1016/j.ctim.2024.103023. PMID: 38232905

[9] Hediger K Wagner J Künzi P Haefeli A Theis F Grob C . Effectiveness of animal-assisted interventions for children and adults with post-traumatic stress disorder symptoms: a systematic review and meta-analysis. Eur J Psychotraumatol. (2021) 12:1879713. doi: 10.1080/20008198.2021.1879713. PMID: 34377357 PMC8330800

[10] Jones MG Rice SM Cotton SM . Incorporating animal-assisted therapy in mental health treatments for adolescents: a systematic review of canine assisted psychotherapy. PloS One. (2019) 14:e0210761. doi: 10.1371/journal.pone.0210761. PMID: 30653587 PMC6336278

[11] Maber-Aleksandrowicz S Avent C Hassiotis A . A systematic review of animal-assisted therapy on psychosocial outcomes in people with intellectual disability. Res Dev Disabil. (2016) 49-50:322–38. doi: 10.1016/j.ridd.2015.12.005. PMID: 26773215

[12] Bert F Gualano MR Camussi E Pieve G Voglino G Siliquini R . Animal assisted intervention: a systematic review of benefits and risks. Eur J Integr Med. (2016) 8:695–706. doi: 10.1016/j.eujim.2016.05.005. PMID: 32362955 PMC7185850

[13] Souter MA Miller MD . Do animal-assisted activities effectively treat depression? A meta-analysis. Anthrozoös. (2007) 20:167–80. doi: 10.2752/175303707X207954

[14] Nimer J Lundahl B . Animal-assisted therapy: a meta-analysis. Anthrozoös. (2007) 20:225–38. doi: 10.2752/089279307X224773

[15] Dixon D Jones C Green R . Understanding the role of the animal in animal-assisted therapy: a qualitative study. Complementary Therapies Clin Pract. (2025) 60:101983. doi: 10.1016/j.ctcp.2025.101983. PMID: 40245492

[16] Berget B Braastad BO . Animal-assisted therapy with farm animals for persons with psychiatric disorders. Ann Ist Super Sanita. (2011) 47:384–90. doi: 10.4415/Ann_11_04_10. PMID: 22194073

[17] Barak Y Savorai O Mavashev S Beni A . Animal-assisted therapy for elderly schizophrenic patients: a one-year controlled trial. Am J Geriatr Psychiatry. (2001) 9:439–42. doi: 10.1097/00019442-200111000-00013. PMID: 11739071

[18] Szewczyk D Fiega J Michalska M Żurek U Lubaszka Z Sikorska E . Therapeutic role of animals: a comprehensive literature review on the prevalent forms and species in animal-assisted interventions. J Education Health Sport. (2023) 45:215–35. doi: 10.12775/JEHS.2023.45.01.015

[19] Wagner C Grob C Hediger K . Specific and non-specific factors of animal-assisted interventions considered in research: a systematic review. Front Psychol. (2022) 28:931347. doi: 10.3389/fpsyg.2022.931347. PMID: 35837630 PMC9274084

[20] IAHAO International Association of Human-Animal Interaction Organizations . IAHAIO International Guidelines on Care, Training and Welfare Requirements for Farm Animals Involved in Animal-Assisted Interventions 2021 (2021). Available online at: https://iahaio.org/wp/wp-content/uploads/2021/09/publication-fa-final-iahaio-guidelines-for-farm-animals-involved-in-aai.pdf (Accessed August 02, 2023).

[21] Marino L Merskin D . Intelligence, complexity, and individuality in sheep. Anim Sentience. (2019) 25:1. doi: 10.51291/2377-7478.1374

[22] Schramm E Breuninger C Wohlfarth R Elsaesser M Piosczyk H Fangmeier T . Effectiveness of nature- and animal assisted mindfulness for relapse prevention in depressed patients with a history of childhood maltreatment. Front Psychiatry. (2022) 13:899318. doi: 10.3389/fpsyt.2022.899318. PMID: 35911224 PMC9329652

[23] Pedersen I Martinsen EW BergetB Braastad BO . Farm animal-assisted intervention for people with clinical depression: a randomized controlled trial. Anthrozoös. (2012) 25:149–60. doi: 10.2752/175303712X13316289505260

[24] Berget B Ekeberg O Braastad BO . Animal-assisted therapy with farm animals for persons with psychiatric disorders: effects on self-efficacy, coping ability and quality of life, a randomized controlled trial. Clin Pract Epidemiol Ment Health. (2008) 4:9. doi: 10.1186/1745-0179-4-9. PMID: 18405352 PMC2323374

[25] Berget B Ekeberg Ø Pedersen I Braastad BO . Animal-assisted therapy with farm animals for persons with psychiatric disorders: effects on anxiety and depression, a randomized controlled trial. Occup Ther Ment Health. (2011) 27:50–64. doi: 10.1080/0164212x.2011.543641. PMID: 41909888

[26] Schmid P Nauss C Jauch-Ederer C Prinz P Tschöke S Uhlmann C . Can sheep help to improve positive emotions, mindfulness and self-efficacy? - a pilot study of animal-assisted intervention as an enhanced CBT-based therapy for substance use disorders. Front Psychiatry. (2024) 15:1432679. doi: 10.3389/fpsyt.2024.1432679. PMID: 39473916 PMC11518790

[27] Schmid P Nauss C Jauch-Ederer C Prinz P Kordeuter AL Tschöke S . The sheep did it again: replication of animal-assisted treatment in psychiatric inpatients with substance use disorder and borderline personality disorder in a randomized controlled trial. Healthcare. (2025) 13:2808. doi: 10.3390/healthcare13212808. PMID: 41228175 PMC12609190

[28] Pile V Williamson G Saunders A Holmes EA Lau JYF . Harnessing emotional mental imagery to reduce anxiety and depression in young people: an integrative review of progress and promise. Lancet Psychiatry. (2021) 8:836–52. doi: 10.1016/S2215-0366(21)00195-4. PMID: 34419188

[29] Hagger MS Conroy D . Imagery, visualization, and mental simulation interventions. In: Hagger MS Cameron LD Hamilton K Hankonen N Lintunen T , editors.The Handbook of Behavior Change. Cambridge Handbooks in Psychology. Cambridge: Cambridge University Press (2020). p. 479–94.

[30] Hoffmann AO Lee AH Wertenauer F Ricken R Jansen JJ Gallinat J . Dog-assisted intervention significantly reduces anxiety in hospitalized patients with major depression. Eur J Integr Med. (2009) 1:145–8. doi: 10.1016/j.eujim.2009.08.002. PMID: 41936479

[31] IAHAIO . IAHAIO White Paper 2014, Updated for 2018. In: The IAHAIO Definitions for Animal Assisted Intervention and Guidelines for Wellness of Animals Involved in AAI IAHAIO International Association of Human-Animal Interaction Organizations (2018). Available online at: http://pat.org.za/wp-content/uploads/2021/04/IAHAIO-WHITE-PAPER-TASK-FORCE-FINAL-REPORT_2018.pdf (Accessed August 02, 2023).

[32] Wohlfarth R Sandstedt L . Animal Assisted Activities with Dogs. Guideline for Basic Requirements & Knowledge. Warsaw: Publishing House of Janusz Korczak Pedagogical University in Warsaw (2016).

[33] Wohlfarth R Olbrich E . Qualitätsentwicklung und Qualitätssicherung in der Praxis tiergestützter Interventionen. Zürich: ESAAT, ISAAT (2014).

[34] Tierärztliche Vereinigung für Tierschutz e.V . Nutzung von Tieren im soziale Einsatz Merkblatt Nr. 131.11 Schafe. Belm: TVT Tierärztliche Vereinigung für Tierschutz e.V. (2011). Available online at: https://www.tierschutz-tvt.de/alle-merkblaetter-undstellungnahmen/#c304 (Accessed March 03, 2022).

[35] Tierärztliche Vereinigung für Tierschutz e.V . Tiere im sozialen Einsatz Merkblatt Nr. 131 (Allgemeine Grundsätze). Belm (2021). Available online at: https://www.tierschutz-tvt.de/alle-merkblaetterund-stellungnahmen/#c304 (Accessed March 03, 2022).

[36] Brelsford VL Dimolareva M Gee NR Meints K . Best practice standards in animal-assisted interventions: how the LEAD risk assessment tool can help. Animals. (2020) 10:974. doi: 10.3390/ani10060974. PMID: 32503309 PMC7341237

[37] Spielberger CD Gorsuch RL Lushene R Vagg PR Jacobs GA . Manual for the State-Trait Anxiety Inventory. Palo Alto, CA: Consulting Psychologists Press (1983).

[38] Julian LJ . Measures of anxiety: state-trait anxiety inventory (STAI), beck anxiety inventory (BAI), and hospital anxiety and depression scale-anxiety (HADS-A). Arthritis Care Res (Hoboken). (2011) 63 Suppl 11:467–72. doi: 10.1002/acr.20561. PMID: 22588767 PMC3879951

[39] Walach H Buchheld N Buttenmüller V Kleinknecht N Schmidt S . Measuring mindfulness-the Freiburg Mindfulness Questionnaire. Construction, validation, short version. J Medit Med Res. (2003) 3:97–8.

[40] Walach H Sauer S Kohls N Rose N Schmidt S . Freiburg mindfulness inventory (FMI) short form and revised form (FMI-13R) - norm scores and psychometrics in a representative German sample. BMC Psychol. (2025) 13:1328. doi: 10.1186/s40359-025-03671-3. PMID: 41299799 PMC12670850

[41] Beierlein C Kemper C Kovaleva A Rammstedt B . Kurzskala zur Erfassung allgemeiner Selbstwirksamkeitserwartungen (ASKU). Methoden Daten Analysen (mda). (2013) 7:251–78. doi: 10.12758/mda.2013.014

[42] Priebe S Watzke S Hansson L Burns T . Objective social outcomes index (SIX): a method to summarize objective indicators of social outcomes in mental health care. Acta Psychiatr Scand. (2008) 118:57–63. doi: 10.1111/j.1600-0447.2008.01217.x. PMID: 18582348

[43] Field A . Discovering statistics using SPSS. London: Sage Publications Ltd (2005).

[44] Cohen J . Statistical Power Analysis for the Behavioral Sciences. New York: Academic Press (1988).

